# Molecular Characterization of the Mouse Superior Lateral Parabrachial Nucleus through Expression of the Transcription Factor Runx1

**DOI:** 10.1371/journal.pone.0013944

**Published:** 2010-11-11

**Authors:** Chrissandra J. Zagami, Stefano Stifani

**Affiliations:** Centre for Neuronal Survival, Montreal Neurological Institute, McGill University, Montreal, Quebec, Canada; University of Auckland, New Zealand

## Abstract

**Background:**

The ability to precisely identify separate neuronal populations is essential to the understanding of the development and function of different brain structures. This necessity is particularly evident in regions such as the brainstem, where the anatomy is quite complex and little is known about the identity, origin, and function of a number of distinct nuclei due to the lack of specific cellular markers. In this regard, the gene encoding the transcription factor Runx1 has emerged as a specific marker of restricted neuronal populations in the murine central and peripheral nervous systems. The aim of this study was to precisely characterize the expression of *Runx1* in the developing and postnatal mouse brainstem.

**Methods and Principal Findings:**

Anatomical and immunohistochemical studies were used to characterize mouse *Runx1* expression in the brainstem. It is shown here that *Runx1* is expressed in a restricted population of neurons located in the dorsolateral rostral hindbrain. These neurons define a structure that is ventromedial to the dorsal nucleus of the lateral lemniscus, dorsocaudal to the medial paralemniscal nucleus and rostral to the cerebellum. *Runx1* expression in these cells is first observed at approximately gestational day 12.5, persists into the adult brain, and is lost in knockout mice lacking the transcription factor Atoh1, an important regulator of the development of neuronal lineages of the rhombic lip. *Runx1*-expressing neurons in the rostral hindbrain produce cholecystokinin and also co-express members of the Groucho/Transducin-like Enhancer of split protein family.

**Conclusion:**

Based on the anatomical and molecular characteristics of the *Runx1*-expressing cells in the rostral hindbrain, we propose that *Runx1* expression in this region of the mouse brain defines the superior lateral parabrachial nucleus.

## Introduction

The brainstem is composed of a multitude of separate nuclei involved in the control and integration of key somatic and autonomic processes [1]. Establishing the origin, identity and mechanisms involved in the development of individual brainstem nuclei has been difficult due to the anatomical complexities of this brain region during development [2]. Thanks to recent technical advances, the identification of various molecular determinants involved in brainstem neuronal specification is now escalating [2]. For instance, the basic-helix-loop-helix transcription factor, Atoh1 (atonal homolog 1), has been shown to be essential for the generation of a number of brainstem neuronal populations derived from the rhombic lip (RL), which is the dorsal-most proliferative neuroepithelium of the developing hindbrain [3], [4]. In turn, such regulators of cell fate can be used as specific molecular markers to further improve our understanding of the development of brainstem neurons.

Another example of a neuronal subtype-specific transcription factor is Runx1 (Runt-related transcription factor 1). Like the two other members of the mammalian Runt-related protein family, Runx1 acts as a context-dependent transcriptional activator or repressor [5], [6]. In the developing murine olfactory epithelium, Runx1 is expressed in mitotic olfactory sensory neuron precursors where it is involved in promoting proliferation [7]. In other neuronal lineages investigated to date, Runx1 is expressed exclusively in post-mitotic neurons and plays important roles in phenotype specification and axonal targeting. For instance, in sensory neurons of the dorsal root ganglia (DRG), Runx1 is essential for the correct specification of the nociceptor subtype and the regulation of axonal outgrowth and targeting [8]–[12]. In the cervical spinal cord, Runx1 is expressed in restricted subpopulations of motor neurons, where it is important for the consolidation of motor neuron developmental programs, including the persistent suppression of interneuron-specific genes [13].

The analysis of *Runx1* expression in neuronal cells other than specific subpopulations of sensory and motor neurons remains incomplete. The aim of the present study was to characterize the identity of a select group of *Runx1*-expressing cells in the brainstem. Here we provide evidence that *Runx1* expression defines a population of post-mitotic neurons located in a dorsolateral position of the rostral hindbrain. These neuronal cells are derived from an *Atoh1*-dependent lineage and likely use cholecystokinin (CCK) as a neurotransmitter or neuromodulator. We propose that these *Runx1*-expressing cells comprise neurons of the superior lateral parabrachial nucleus.

## Results

### *Runx1* expression in a restricted population of postmitotic neurons in a dorsolateral position of the rostral hindbrain

**Figure 1:**
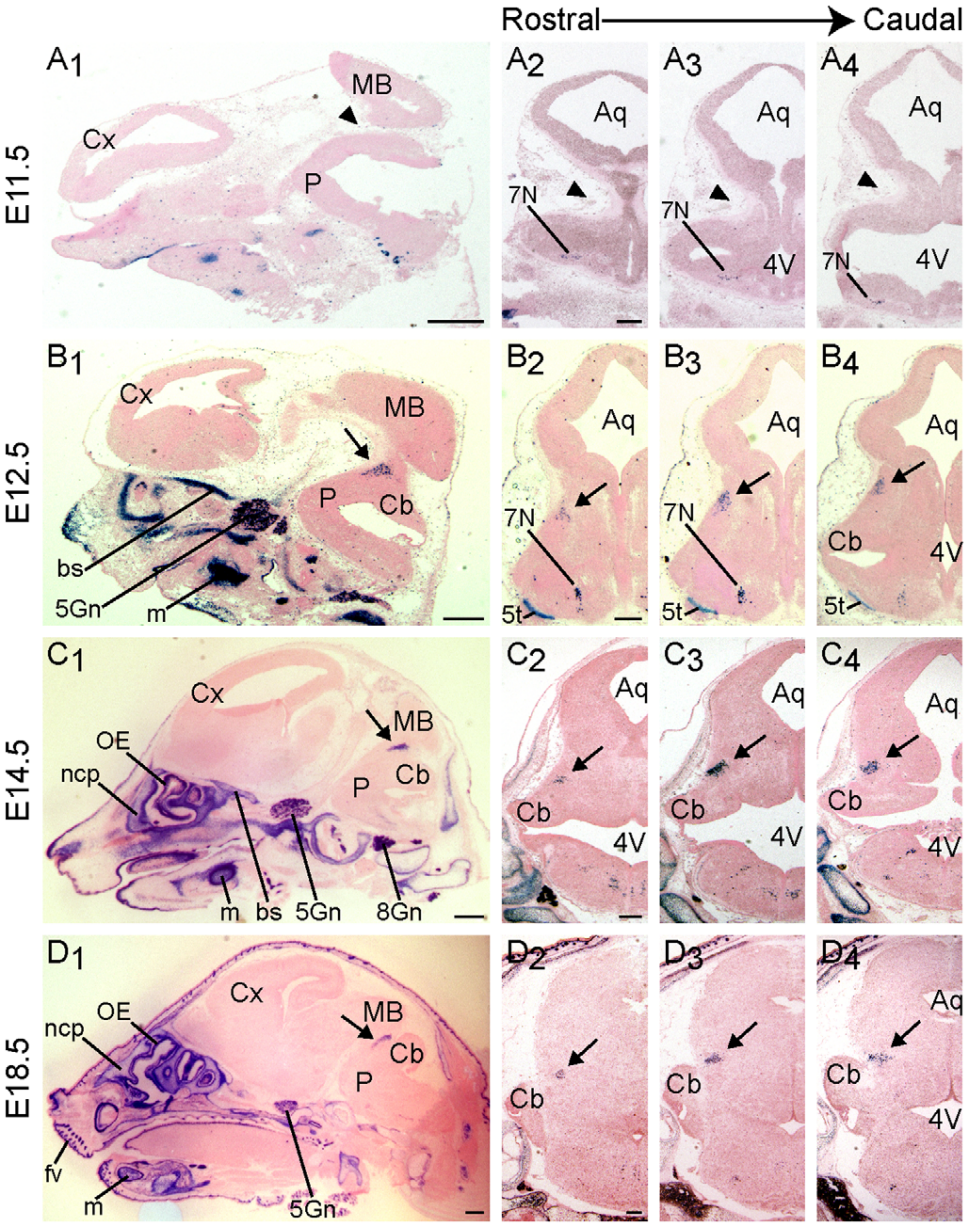
Analysis of ß-gal expression in the dorsolateral rostral hindbrain of *Runx1* ^lacZ/**+**^ mouse embryos. Sagittal (column 1) or coronal (columns 2–4; from rostral to caudal positions, respectively) sections from *Runx1* ^lacZ/+^ embryos collected at the indicated stages were subjected to X-gal staining to detect the activity of the ß-gal protein. (A) No ß-gal activity is observed at E11.5, when the dorsal rostral hindbrain does not as yet appear to have fully developed (arrowheads). (B-D) ß-gal activity (blue color) becomes detectable from E12.5 in the dorsolateral rostral hindbrain (B; arrows) and continues throughout embryonic development (C-D; arrows). In this and all subsequent figures, sagittal sections are oriented with dorsal at the top and rostral to the left and coronal sections are oriented with dorsal at the top and lateral to the left. Abbreviations: 4V, fourth ventricle; 5Gn, trigeminal ganglion; 5t, spinal trigeminal tract; 7N, nucleus of the seventh (facial) nerve; 8Gn, vestibulocochlear ganglion; Aq, aqueduct; bs, basisphenoid chondrogenic centre; Cb, cerebellum; Cx, cortex; fv, follicles of the vibrissae; m, Meckel's cartilage; MB, midbrain; ncp, nasal capsule; OE, olfactory epithelium; P, pons. Scale bars: column 1 = 400 µm, columns 2–4 = 200 µm.

**Figure 2:**
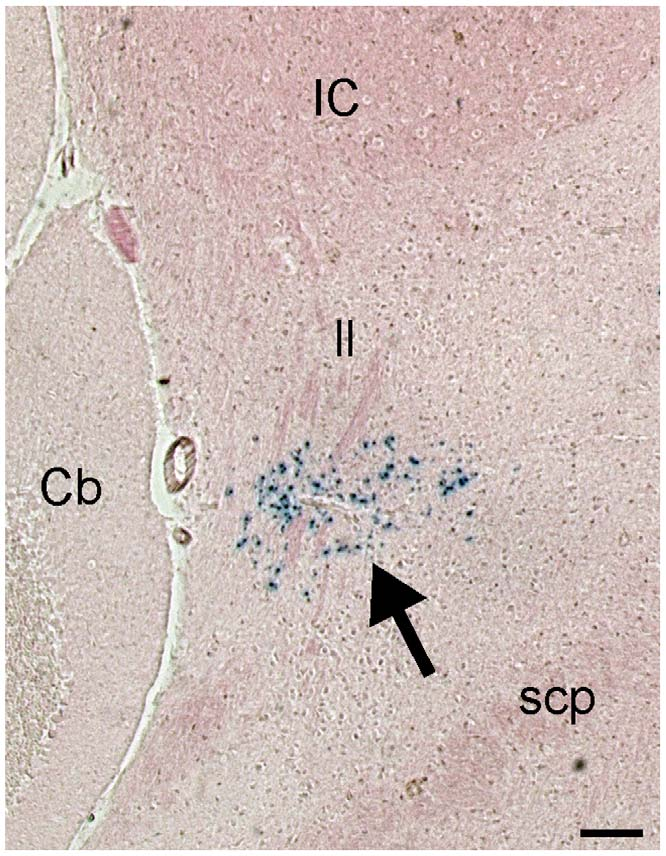
Detection of ß-gal activity in the dorsolateral rostral hindbrain of adult *Runx1* ^lacZ/**+**^ mice. Coronal section from a *Runx1* ^lacZ/+^ adult mouse brain subjected to X-gal staining to detect the activity of the ß-gal protein (blue color; arrow). Abbreviations: Cb, cerebellum; IC, inferior colliculus; ll, lateral lemniscus; scp, superior cerebellar peduncle. Scale bar = 200 µm.

To characterize *Runx1* expression in the developing brainstem, we used a previously described *Runx1* ^lacZ/+^ knock-in mouse line in which the expression of ß-galactosidase (ß-gal) faithfully replicates Runx1 transcript and protein expression [13]–[16]. Analysis of ß-gal enzyme activity did not reveal the presence of any ß-gal^+^ cells in the dorsal area of the rostral hindbrain of embryonic day 11.5 (E11.5) *Runx1* ^lacZ/+^ embryos (Fig. 1A). However, by E12.5 a previously uncharacterized group of ß-gal^+^ cells was visible in a dorsolateral position of the rostral hindbrain (Fig. 1B), near the border with the midbrain, suggesting that *Runx1* expression becomes activated in this region between E11.5 and E12.5. Cells expressing ß-gal were detected in the dorsolateral rostral hindbrain throughout embryonic development (Fig. 1C and 1D) and expression persisted into adulthood (postnatal day P50–P70) (Fig. 2 and supporting information Fig. S1).

Analysis in adult *Runx1* ^lacZ/+^ mice demonstrated ß-gal^+^ cells located within a roughly triangular group of fairly dense cells in the dorsolateral rostral hindbrain (Fig. S1). More specifically, this group of ß-gal^+^ cells was anatomically situated amongst, and medial to, fibers of the lateral lemniscus (ll), ventral to the inferior colliculus and dorsolateral to the superior cerebellar peduncle (Fig. 2). Examination of the embryonic development of ß-gal^+^ cells in both sagittal and coronal planes through the dorsolateral rostral hindbrain (Fig. 1) revealed that this group of cells becomes increasingly more dorsal from its rostral to caudal aspect. In the coronal plane, the group of ß-gal^+^ cells appeared triangular in shape, particularly at the more rostral extent. In the sagittal plane, the ß-gal^+^ cells were anatomically located rostral to the cerebellum. Using double-label immunofluorescence staining for ß-gal and tuberoinfundibular peptide of 39 residues (TIP39), a previously characterized marker of the medial paralemniscal nucleus (MPL) [17], [18], we found no overlap of TIP39 and ß-gal expression (Fig. S2). Instead, the group of ß-gal^+^ neurons was located dorsocaudal to the TIP39^+^ MPL. Thus, along the rostrocaudal axis, the *Runx1*-expressing neurons in the dorsolateral rostral hindbrain are positioned between the MPL and the cerebellum.

**Figure 3:**
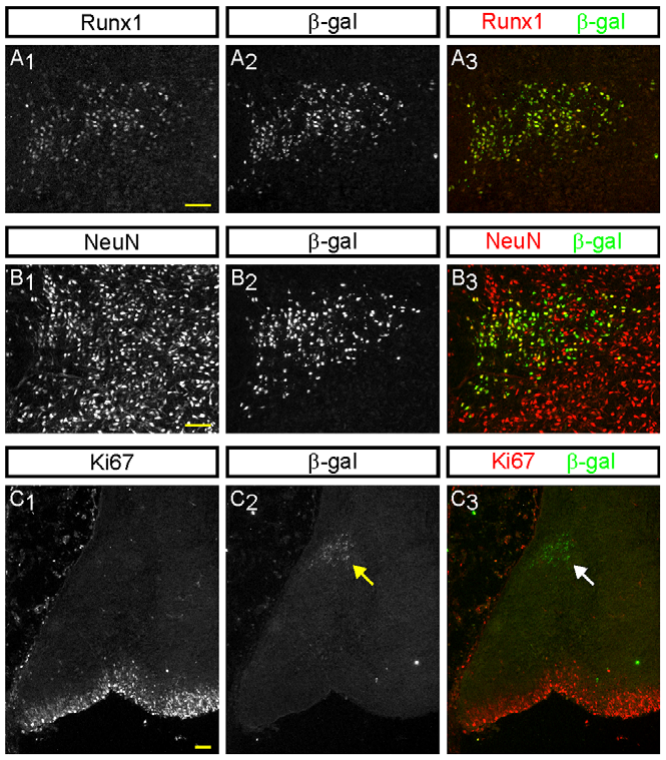
Expression of ß-gal and Runx1 in postmitotic neurons of the dorsolateral rostral hindbrain of *Runx1* ^lacZ/**+**^ mouse embryos. Double-label immunofluorescence staining of the dorsolateral rostral hindbrain within coronal sections from E18.5 *Runx1* ^lacZ/+^ embryos demonstrates co-expression of ß-gal with either Runx1 (A) or NeuN (B). (C) No overlap of ß-gal (arrows) and Ki67 was observed at E12.5, when many proliferating cells are present at a distance from the ß-gal^+^ cells. Scale bars = 50 µm.

Double-label immunofluorescence analysis demonstrated co-expression of ß-gal and Runx1 at various developmental stages in the dorsolateral rostral hindbrain of *Runx1* ^lacZ/+^ mice, indicating that ß-gal expression indeed reflected that of the Runx1 protein (Fig. 3A). In addition, the ß-gal^+^ cells of this region were immunoreactive for the general neuronal markers NeuN and MAP2 (Fig. 3B) (see also Fig. S3), but not for the cell proliferation marker Ki67 (Fig. 3C), indicating that they correspond to postmitotic neurons. Taken together, these data show that *Runx1* is expressed from E12.5 to adulthood in a group of cells that comprise a population of postmitotic neurons within the dorsolateral rostral hindbrain.

### Location of *Runx1*-expressing neurons in the superior lateral subnucleus of the parabrachial complex

The location of the newly identified group of *Runx1*-expressing neurons in the dorsolateral rostral hindbrain suggested that these cells might be situated within a nucleus of the ll or the parabrachial nucleus (PB). The ll is composed of various ascending fibers of the auditory system embedded in which are three nuclei, designated the dorsal (DLL), intermediate (ILL) and ventral (VLL) nuclei of the ll [19]. The PB, which is divided into several lateral (LPB) and medial (MPB) subnuclei and also includes the ventrolateral Kölliker-Fuse subnucleus (KF) [20], plays diverse roles in various systems, including the respiratory, nociceptive and vestibular systems [21]–[23].

**Figure 4:**
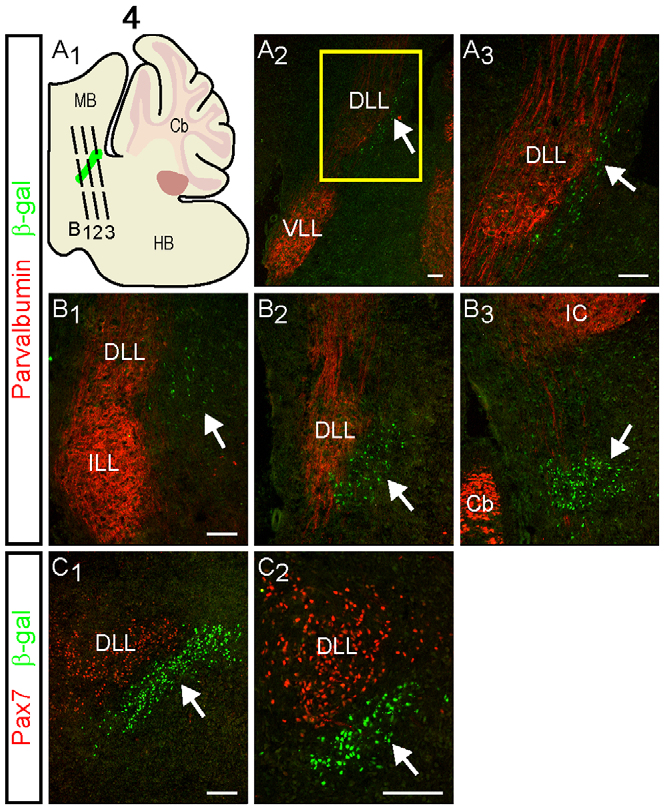
*Runx1*-expressing cells are not located in the nuclei of the lateral lemniscus. Double-labeling analysis of ß-gal and PV (A and B) or Pax7 (C) expression in sagittal (A and C_1_) or coronal (B and C_2_) sections of E18.5 *Runx1* ^lacZ/+^ mouse embryos. (A_1_) Schematic of the hindbrain in the sagittal plane demonstrating the location of the group of ß-gal^+^ neurons in green. The boxed area in A_2_ is shown magnified in A_3_. (B_1_–B_3_) Coronal hemisections are shown from rostral to caudal as indicated by the approximate levels shown in A_1_. The expression of ß-gal (arrows) does not overlap with that of the PV^+^ DLL, ILL or VLL, nor the Pax7^+^ DLL. Instead, the group of ß-gal^+^ cells is located ventromedial to the PV^+^ and Pax7^+^ DLL. Abbreviations: Cb, cerebellum; DLL, dorsal nucleus of the lateral lemniscus; IC, inferior colliculus; ILL, intermediate nucleus of the lateral lemniscus; VLL, ventral nucleus of the lateral lemniscus. Scale bars = 50 µm.

To begin to examine the identity of the *Runx1*-expressing neurons in the dorsolateral rostral hindbrain, we compared the expression of ß-gal to that of the calcium-binding protein, parvalbumin (PV), on sections from P10 *Runx1* ^lacZ/+^ mice. At this age, PV is expressed in all the nuclei of the ll, which is not the case in embryos [24]. No overlap of ß-gal and PV was detected; instead, the ß-gal^+^ cells were observed to lie ventral to the PV^+^ DLL (Fig. 4A and 4B). None of the ß-gal^+^ cells corresponded to PV^+^ cells of the VLL, which is situated at a more rostroventral location (Fig. 4A_2_). At the most rostral extent of the group of ß-gal^+^ cells, where both the ILL and DLL can be observed in the coronal plane but only few ß-gal^+^ cells are present, the latter were located medially to the ventral half of the DLL (Fig. 4B_1_). More caudally, where the DLL, but not ILL, was present, the ß-gal^+^ cells were visible along the ventromedial border of the PV^+^ DLL (Fig. 4B_2_). At the most caudal extent of the group of ß-gal^+^ cells, the PV^+^ nuclei of the ll were no longer present although a few PV^+^ fibers of the ll could still be observed amongst the group of ß-gal^+^ cells (Fig. 4B_3_).

The position of the group of ß-gal^+^ cells relative to the DLL in E18.5 *Runx1* ^lacZ/+^ embryos was confirmed through immunostaining for ß-gal and Pax7, a transcription factor previously reported to mark the DLL [25]. This analysis revealed no detectable overlap of the two proteins; instead, the group of ß-gal^+^ cells was located ventromedial to the Pax7^+^ cells of the DLL (Fig. 4C). Thus, immunostaining for both PV and Pax7 indicates that the ß-gal^+^ neurons are not located within the nuclei of the ll but lie medially to the DLL.

**Figure 5:**
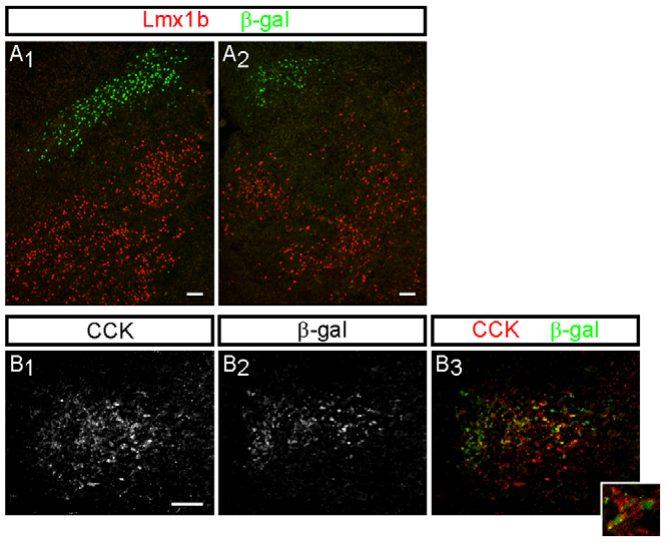
Expression of ß-gal, Lmx1b and CCK in the dorsolateral rostral hindbrain of E18.5 *Runx1* ^lacZ/**+**^ mouse embryos. (A) Double-label immunostaining for ß-gal and Lmx1b demonstrates no overlapping expression of these proteins in either the sagittal (column 1) or coronal (column 2) planes. Instead, the group of ß-gal^+^ cells is located dorsal to the Lmx1b^+^ cells of the PB. (B) Overlapping immunoreactivity for CCK (B_1_) and ß-gal (B_2_) within the same coronal region of the dorsolateral rostral hindbrain (B_3_) and within some cells (inset). Scale bars = 50 µm.

Fairly rostrally within the PB of the adult rat, the LPB is bordered laterally by the DLL [20]. In particular, the superior lateral subnucleus of the PB (LPBS) shares several anatomical similarities with the group of *Runx1*-expressing neurons in the dorsolateral rostral hindbrain. Such similarities include a triangular contour, a progressively more dorsal position along the rostrocaudal axis, and a position rostral to the cerebellum in the sagittal plane [20]. Moreover, the LPBS extends dorsally above the rest of the PB [20]. The LIM homeobox transcription factor 1 beta (Lmx1b) has been reported to be expressed in both the LPB and MPB, as well as in the KF of postnatal and adult mice [26]. We therefore compared the expression of ß-gal and Lmx1b in E18.5 *Runx1* ^lacZ/+^ embryos. The expression of ß-gal did not coincide with that of Lmx1b and the group of ß-gal^+^ neurons was positioned mostly dorsal to Lmx1b^+^ cells (Fig. 5A).

A molecular marker which is relatively specific for the LPBS is the gene encoding the neuropeptide CCK. *Preprocholecystokinin* (*ppCCK*) mRNA is robustly expressed in the LPBS compared to other subnuclei of the PB in which the *ppCCK* transcript is only expressed in moderate to few numbers of neurons [27]. Similarly, CCK-immunoreactive neurons have also been observed in the LPBS of the rat [28]. To determine whether the *Runx1*-expressing neurons in the dorsolateral rostral hindbrain might be part of the LPBS, we performed double-label immunofluorescence staining for ß-gal and CCK on sections from E18.5 *Runx1* ^lacZ/+^ mice. The group of ß-gal^+^ cells in this hindbrain area was localized within a region of CCK immunoreactivity and some, but not all, ß-gal^+^ cells appeared to express CCK (Fig. 5B). Taken together with their rostral position, these data suggest that the group of *Runx1*-expressing neurons in the dorsolateral rostral hindbrain is located within the LPBS and at least a subset of these neurons likely express CCK but not Lmx1b.

### Loss of *Runx1* expression in neurons of the superior lateral parabrachial nucleus in *Atoh1-*deficient mice

**Figure 6:**
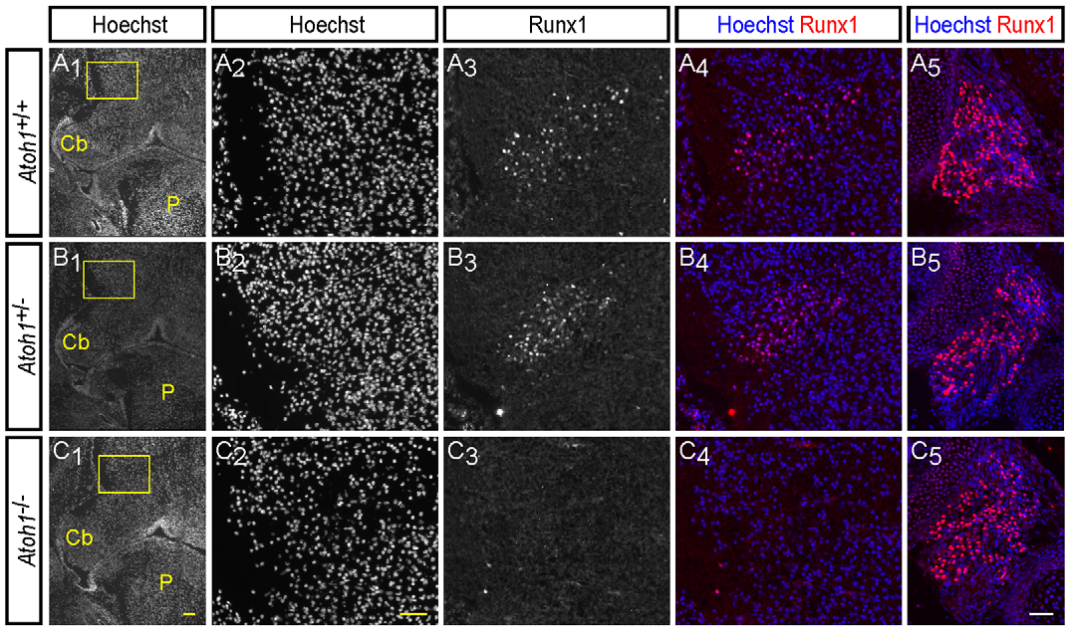
Runx1 protein expression in the dorsolateral rostral hindbrain of *Atoh1* knockout embryos. Analysis of Runx1 expression is shown in coronal sections from E14.5 *Atoh1* ^+/+^ (A), *Atoh1* ^+/–^ (B) and *Atoh1* ^–/–^ (C) mouse embryos. Hoechst staining is also shown. The boxed area in column 1 indicates the region magnified in columns 2–4. Staining from column 2 and column 3 is shown merged in column 4. Column 5 depicts Runx1 expression in a vestibulocochlear ganglion of the same section depicted in column 1. Runx1 is expressed in both the dorsolateral rostral hindbrain and vestibulocochlear ganglia of *Atoh1* ^+/+^ and *Atoh1* ^+/–^ embryos, but only in the vestibulocochlear ganglia, and not the dorsolateral rostral hindbrain, of *Atoh1* ^–/–^ embryos. Abbreviations: Cb, cerebellum; P, pons. Scale bars: column 1 = 100 µm, columns 2–5 = 50 µm.

Certain PB nuclei have previously been reported to be derived from *Atoh1*-dependent progenitors of the RL [3], [4]. To determine whether *Runx1*-expressing neurons of the LPBS are derived from *Atoh1*-expressing progenitors, we compared the expression of the Runx1 protein in E14.5 *Atoh1* ^–/–^ embryos and their *Atoh1* ^+/+^ and *Atoh1* ^+/–^ littermates [29]. Runx1^+^ cells were detected in the LPBS in sections from *Atoh1* ^+/+^ (Fig. 6A_1–4_) and *Atoh1* ^+/–^ (Fig. 6B_1–4_) littermates and there did not appear to be a difference in Runx1 immunoreactivity between those embryos. In agreement with previous studies [16], Runx1 expression was also observed in the vestibulocochlear ganglia of *Atoh1* ^+/+^ (Fig. 6A_5_) and *Atoh1* ^+/–^ (Fig. 6B_5_) embryos. In contrast, no detectable Runx1 expression was observed in the LPBS of *Atoh1* ^–/–^ littermates (Fig. 6C_1–4_), even though Runx1 immunoreactivity was readily observable in cells of the vestibulocochlear ganglia within the same sections (Fig. 6C_5_). The absence of Runx1 expression in the LPBS of *Atoh1* ^–/–^ mice provides evidence that the *Runx1*-expressing neurons of this region are derived from *Atoh1*-expressing progenitors.

### Monoamine and amino acid neurotransmitter expression in the region of the superior lateral parabrachial nucleus

**Figure 7:**
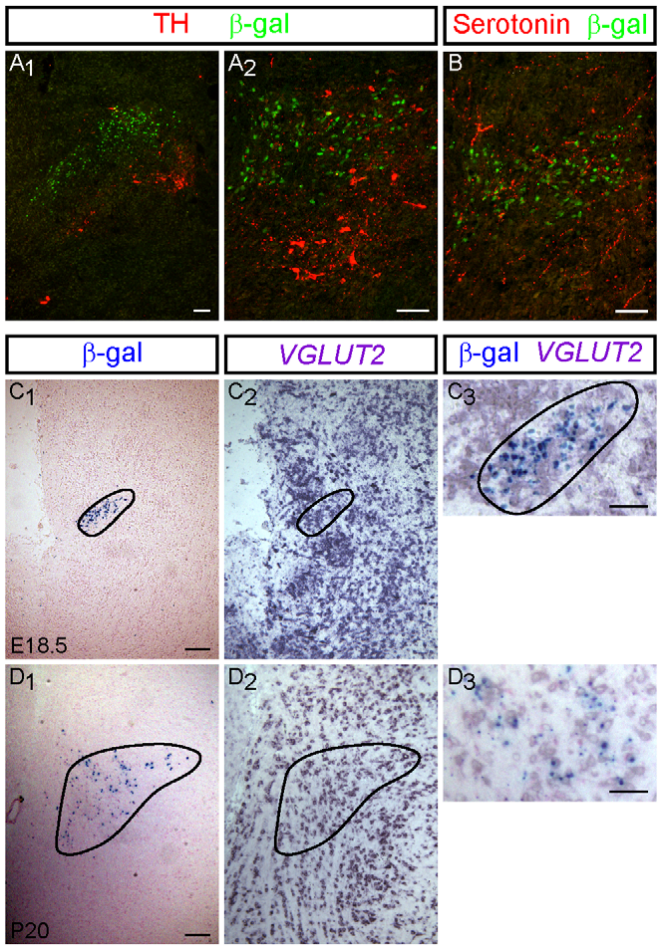
Neurotransmitter phenotype of *Runx1*-expressing neurons in the dorsolateral rostral hindbrain. (A) ß-gal and TH expression in sagittal (A_1_) and coronal (A_2_) sections from E18.5 *Runx1* ^lacZ/+^ mouse embryos. (B) ß-gal and serotonin expression in the coronal plane of the dorsolateral rostral hindbrain of E18.5 *Runx1* ^lacZ/+^ mouse embryos. The non-overlapping expression of ß-gal and neither TH nor serotonin demonstrates that the ß-gal^+^ cells are not catecholaminergic or serotonergic. However, the ß-gal^+^ cells are located mostly dorsal to TH^+^ cells and are partly situated within a region containing TH and serotonin immunoreactivity likely marking fiber tracts. (C–D) Comparison of X-gal staining (C_1_ and D_1_; visualizing the region of ß-gal activity) and *VGLUT2* mRNA expression (C_2_ and D_2_) in adjacent serial sections from E18.5 (C) and P20 (D) *Runx1* ^lacZ/+^ mice. Images of adjacent sections shown in C_1_ and C_2_, and D_1_ and D_2_, were merged and are shown magnified in C_3_ and D_3_, respectively. Scale bars: A, B, C_3_ and D_3_ = 50 µm, C_1_/C_2_ and D_1_/D_2_ = 100 µm.

Neuropeptides such as CCK often act as co-transmitters. To begin to determine whether the *Runx1*-expressing neurons of the LPBS might also express another neurotransmitter, we used E18.5 *Runx1* ^lacZ/+^ embryos to compare the expression of ß-gal to that of tyrosine hydroxylase (TH) or serotonin. The ß-gal^+^ neurons of the LPBS did not co-express either TH (Fig. 7A) or serotonin (Fig. 7B), suggesting that they are not catecholaminergic or serotonergic neurons, respectively. We noticed that the caudal extent of the group of ß-gal^+^ cells was located mostly dorsal to, but was also interspersed with, TH immunoreactivity. The ß-gal^+^ cells were also interspersed with some serotonin immunoreactivity. These observations are consistent with previous studies demonstrating TH-containing fibers and some cell bodies in the dorsal LPB, as well as serotonin fibers [30].

Neuronal lineages of the *Atoh1*-expressing progenitors of the RL are predominantly glutamatergic [4], [31]. We examined whether ß-gal^+^ cells of the dorsolateral rostral hindbrain might be glutamatergic using *in situ* hybridization to label the glutamatergic marker *vesicular glutamate transporter 2* (*VGLUT2*) in E18.5 and P20 *Runx1* ^lacZ/+^ mice. *VGLUT2* mRNA was demonstrated in the region of ß-gal activity in the dorsolateral rostral hindbrain of both E18.5 (Fig. 7C) and P20 (Fig. 7D) animals, suggesting that ß-gal^+^ cells of the dorsolateral rostral hindbrain could be glutamatergic. In potential agreement with this possibility, we observed VGLUT2 immunoreactivity in the area of ß-gal^+^ cells of the dorsolateral rostral hindbrain of E18.5 *Runx1* ^lacZ/+^ embryos (Fig. S3).

### Expression of Groucho/Transducin-like Enhancer of split proteins in *Runx1*-expressing neurons of the superior lateral parabrachial nucleus

**Figure 8:**
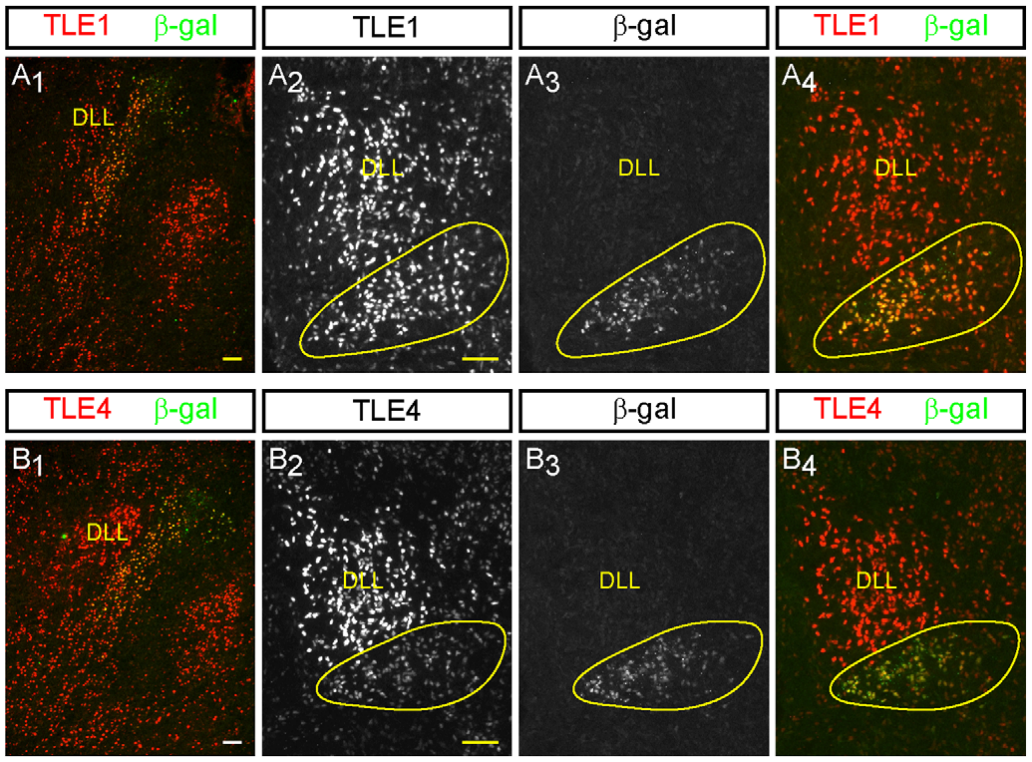
Analysis of ß-gal, TLE1 and TLE4 expression in the dorsolateral rostral hindbrain of E18.5 *Runx1* ^lacZ/**+**^ mouse embryos. Double-labeling immunofluorescence analysis of ß-gal and either TLE1 (A) or TLE4 (B) is shown in the sagittal (column 1) and coronal (columns 2–4) planes. Staining from column 2 and column 3 is shown merged in column 4. TLE1 and TLE4 are both expressed in the DLL as well as in the group of ß-gal^+^ cells located ventromedial to the DLL. Abbreviation: DLL, dorsal nucleus of the lateral lemniscus. Scale bars = 50 µm.

To further characterize the *Runx1*-expressing cells in the LPBS of *Runx1* ^lacZ/+^ embryos, we compared ß-gal expression to that of members of the Groucho/Transducin-like Enhancer of split (Gro/TLE) protein family [32]. Gro/TLEs are transcriptional corepressors that interact with Runx proteins and are selectively recruited by the latter to repress the expression of specific genes in a context-dependent manner [33], [34]. Immunostaining with previously characterized [35], [36] antibodies against two Gro/TLE family members, TLE1 (Fig. 8A) and TLE4 (Fig. 8B), showed that these proteins are expressed in the ß-gal^+^ neurons of the LPBS of E18.5 *Runx1* ^lacZ/+^ mouse embryos. This finding provides further molecular characterization of the LPBS and raises the possibility that Runx1 and Gro/TLE proteins might work together in this brainstem nucleus.

## Discussion

The present study demonstrates the expression of *Runx1* in selected postmitotic neurons of the developing and adult mouse dorsolateral rostral hindbrain. This distinct neuronal population shares various anatomical and molecular characteristics with the LPBS. Like the LPBS [20], the group of *Runx1*-expressing neurons was observed to be bordered laterally by the DLL and caudally by the cerebellum, have a triangular shape, extend dorsally above the rest of the PB and occupy a progressively more dorsal position along the rostrocaudal axis. Molecularly, the *Runx1*-expressing neurons in the dorsolateral rostral hindbrain were situated within a region of CCK immunoreactivity, and several were also shown to express CCK, a fairly specific marker for the LPBS in this area [27]. Moreover, similar to previous reports regarding the PB [3], [4], [30], the *Runx1*-expressing neurons of the dorsolateral rostral hindbrain were found to be derived from *Atoh1*-expressing progenitors and interspersed with TH and serotonin immunoreactivity. In addition to sharing such features with the LPBS, the *Runx1*+ neurons were also found to express Gro/TLE, but not Lmx1b. Thus, we propose that Runx1^+^/CCK^+^/Gro/TLE^+^/Lmx1b^–^ cells in the dorsolateral rostral hindbrain define neurons located within the LPBS.

*Runx1* is expressed in postmitotic neurons of the LPBS from approximately E12.5 and its expression continues at least into young adulthood. However, we cannot exclude the possibility that the expression of Runx1 may end shortly before the last time point tested here, due to the ß-gal protein possibly having a longer half-life than that of Runx1. The postmitotic expression of *Runx1* in neurons of the LPBS is consistent with postmitotic expression of this gene in motor and sensory neurons of the central nervous system (CNS) and peripheral nervous system (PNS), respectively. Within the CNS, *Runx1* is expressed in restricted populations of postmitotic motor neurons in the hindbrain and cervical spinal cord [13], [16]. Similarly, *Runx1* is expressed within the PNS in particular populations of postmitotic trigeminal, vestibular and DRG sensory neurons [8]–[11], [16]. Investigations of those specific neuronal populations suggested roles for Runx1 in the regulation of neuronal subtype specification and axonal targeting [8]–[13]. Based on these previous observations, the postmitotic expression of *Runx1* in neurons of the LPBS and its persistence into adulthood suggests that Runx1 may be involved in the specification and/or maintenance of the identity of these neurons, and/or their target connectivity. Furthermore, the expression of TLE1 and TLE4 in the *Runx1*-expressing neurons of the LPBS may indicate that the role of Runx1 in these cells may, at least in part, be mediated through the recruitment of Gro/TLE proteins and consequent transcriptional repression, although this possibility remains to be verified.

Various components of certain integrated functional networks, including the vestibular, auditory, proprioceptive, interoceptive, arousal and respiratory systems, share a developmental requirement for *Atoh1* [3], [4], [37], [38]. The finding that Runx1 is expressed in neurons of the LPBS presents a possibility that Runx1 could also play a role in certain integrated functional systems. Neurons of the LPBS of the rat have been demonstrated to project to the hypothalamus and predominantly to the ventromedial hypothalamic nucleus (VMH) [20], [28]. The majority of the neurons which project from the LPBS to the VMH contain CCK [28] and, moreover, the VMH contains receptors for CCK [39], [40]. Evidence suggests that the CCK-containing neurons of the LPBS projecting to the VMH are involved in the suppression of food intake. CCK has long been known to suppress food intake [41], [42] and conversely, damage to the VMH [reviewed in 43] and LPB [44] have been reported to lead to hyperphagia and obesity. A separate line of study suggests a link between pain and appetite which involves *CCK*-expressing neurons projecting from the LPBS to the VMH. Through an investigation into the possible mechanisms underlying the common complaint of loss of appetite amongst pain patients, Malick and colleagues [45] demonstrated that noxious stimulation of the dura of rats, a model of migraine pain, resulted in a decrease in food intake. This pain-induced suppression of food intake was associated with an activation of neurons not only in the spinal trigeminal nucleus, but also in the LPBS and VMH. Further analyses revealed that at least a subset of the activated neurons in the LPBS expressed *CCK* mRNA and in the VMH expressed transcripts for CCK receptors. Incidentally, evidence also suggests that noxious information from the spinal cord activates hypothalamic-projecting neurons of the LPBS. Mechanical, thermal and inflammatory noxious stimuli applied to a hindpaw of rats have been demonstrated to activate the LPBS, where the latter two forms of stimuli were expressly shown to activate hypothalamic-projecting neurons of the PB, including from the LPBS [46]–[48]. In addition, noxious chemical stimulation of the rat hindpaw was also reported to activate LPBS neurons, almost all of which expressed *ppCCK* transcripts [27].

Like the PB, Runx1 has also been shown to be involved in the nociceptive network. Runx1 is expressed in nociceptors of DRG [49] and trigeminal ganglia [16]. In DRG, Runx1 is involved in the correct specification of precise nociceptor populations, including regulation of the expression of nociceptor-specific receptors and ion channels [8]–[12], and recent evidence suggests Runx1 may play a similar role in nociceptors of trigeminal ganglia [50]. Moreover, alterations in *Runx1*-expression in DRG cause perturbations in the outgrowth and axonal targeting of nociceptors, as well as in the response to thermal, neuropathic and inflammatory, but not mechanical, pain [8]–[12]. Taken together with these previous findings of the expression and role of Runx1 in DRG and trigeminal ganglia nociceptors, the present demonstration of *Runx1*-expressing neurons in the LPBS might suggest a role for this transcription factor in a functional network involving nociception and regulation of food intake.

In summary, the present findings identify a new group of *Runx1*-expressing neurons in the brain and provide evidence that these neurons are part of the LPBS. The expression of *Runx1* within the neurons of the LPBS will undoubtedly provide both a useful marker and a genetic tool for future investigations into the development and precise functional role of this nucleus.

## Materials and Methods

### Ethics Statement

All animal procedures were conducted in accordance with the guidelines of the Canadian Council for Animal Care and were approved by the Animal Care Committee of the Montreal Neurological Institute of McGill University (animal use protocol No. 5468).

### Mouse Lines and Tissue Preparation

*Runx1* ^lacZ/+^ mice were generated as described previously [15] and the genotype determined by PCR analysis of genomic DNA obtained from tail biopsies and by assaying for ß-gal activity [15], [16]. Mice heterozygous and homozygous for the targeted deletion of *Atoh1* were also generated and genotyped as previously described [29], and were provided by Dr. J. E. Johnson (University of Texas Southwestern Medical Center, TX). For embryonic staging of all animals, the day of appearance of the vaginal plug was considered as E0.5. Staged embryos and postnatal brains were recovered, fixed, and cryostat sections (14 µm) prepared as described previously [16].

### Detection of ß-gal Activity in *Runx1* ^lacZ/+^ Mice

Sections were incubated overnight at 37°C in solution containing 1 mg/ml 5-bromo-4-chloro-3-indolyl- ß-galactopyranoside (X-gal) (Invitrogen, Carlsbad, CA), 5 mM potassium ferricyanide, 5 mM potassium ferrocyanide, 80 mM Na_2_HPO_4_, 20 mM NaH_2_PO_4_, 2 mM MgCl_2_, 0.2% IGEPAL and 0.1% sodium deoxycholate. Following this time, sections were rinsed extensively in phosphate-buffered saline (PBS) and counterstained with eosin before mounting with Fluoromount-G (SouthernBiotech, Birmingham, AL). Images of X-gal stained sections were captured using a Retiga EXi Camera (QImaging, Surrey, BC, Canada) mounted on a Zeiss Axio Imager.M1 microscope (Zeiss, Toronto, ON, Canada) and Northern Eclipse software (Empix Imaging, Inc., Mississauga, ON, Canada).

### Cresyl Violet (Vogt's) Staining for Nissl Substance

Sections were pre-incubated in absolute alcohol for 2 h prior to incubation in 0.02% cresyl echt violet with 0.2 w/v sodium acetate and 0.3% glacial acetic acid for 1 h at room temperature. Following staining, sections were differentiated rapidly in 95% alcohol, dehydrated and cleared through absolute alcohol and xylene and mounted with Permount (Fisher Scientific, Toronto, ON, Canada). Images of cresyl violet stained sections were captured using a Retiga EXi Camera mounted on a Zeiss Axio Imager.M1 microscope and Northern Eclipse software.

### Immunofluorescence

Immunofluorescence staining involving mouse primary antibodies was performed using a Vector Mouse on Mouse Kit (Vector Laboratories, Inc., Burlington, ON, Canada). All other single, double and triple-label immunofluorescence experiments were performed by first blocking non-specific staining with blocking solution containing 1% normal donkey serum and 0.1% Triton X-100 in PBS for 15 min. Sections were then incubated sequentially with primary (2 h) and secondary (1 h) antibodies in blocking solution. The following primary antibodies were used: goat anti-ß-gal (1∶1,000; Biogenesis Inc., Hackensack, NJ), rabbit anti-ß-gal (1∶2,000; Cappel, MP Biomedicals, Solon, OH), mouse anti-Ki67 (1∶500; BD Biosciences Pharmingen, Mississauga, ON, Canada), rabbit anti-PV (1∶5,000; Swant, Bellinzona, Switzerland), mouse anti-Pax7 (1∶50; Developmental Studies Hybridoma Bank, Iowa City, Iowa), chick anti-MAP2 (1∶1,000; GeneTex Inc., Irvine, CA), rabbit anti-serotonin (1∶500) and rabbit anti-CCK (1∶500) (ImmunoStar Inc., Hudson, WI), mouse anti-NeuN (1∶200), rabbit anti-TH (1∶500) and guinea-pig anti-VGLUT2 (1∶2,000) (Millipore Corporation, Temecula, CA), rabbit anti-Runx1 (1∶2,000; a kind gift from Dr. T.M. Jessell, Columbia University, NY), rabbit anti-TIP39 (1∶1,000; a kind gift from Dr. T.B. Usdin, NIMH, MD), guinea-pig anti-Lmx1b (1∶10,000; a kind gift from Dr. C. Birchmeier, Max Delbrück Center for Molecular Medicine, Berlin), and rabbit anti-TLE1 (1∶500) and rabbit-TLE4 (1∶500) [35], [36], [51]. The fluorescent conjugated secondary antibodies used included the Alexa Fluor 488 and 555 series (1∶1,000; Molecular Probes, Invitrogen), as well as the cyanine fluorescent conjugated secondary antibodies of the Cy3 and Cy5 series (1∶500; Jackson ImmunoResearch Laboratories, Inc., West Grove, PA). Digital images of immunofluorescence staining were acquired using a Digital Video Camera (DVC, Austin, TX) attached to a Zeiss Axioskop 2 microscope and Northern Eclipse software. Alternatively, images were captured using a Zeiss LSM 510 confocal microscope with associated Zeiss Enhanced Navigation software.

### *In Situ* Hybridization

Sections were fixed in 4% paraformaldehyde in PBS for 20 min and rinsed three times in PBS. Acetylation was performed in 0.25% acetic anhydride in 1% triethanolamine for 10 min. Sections were rinsed twice in PBS, once in 2x saline-sodium citrate buffer (SSC; 300 mM NaCl, 30 mM sodium citrate, pH 7.0) and prehybridized for 2 h in 50% formamide, 5x Denhardt's solution (0.1% each of Ficoll, polyvinylpyrrolidone, bovine serum albumin), 5x SSC, 250 µg/ml baker's yeast tRNA for 2 h. Hybridization was performed overnight at 60°C using a digoxigenin (DIG)-labeled *VGLUT2* riboprobe kindly provided by Dr. Q. Ma (Harvard Medical School, Boston, MA) [52]. The following day, sections were washed for 5 min each in 5x SSC and 2x SSC at 60°C, for 30 min in 50% formamide in 0.2x SSC at 60°C, and for 5 min each in 0.2x SSC and Tris-buffered saline (TBS; 100 mM Tris, 150 mM NaCl, pH 7.5) at room temperature. Blocking was performed for 1 h in 10% normal goat serum in TBS followed by a 3 h incubation with anti-DIG antibody (1∶3,000) conjugated to alkaline phosphatase (Roche Applied Science, Mannheim, Germany) in TBS. Sections were washed twice in TBS for 15 min, once in solution containing 100 mM Tris, 100 mM NaCl, 5 mM MgCl_2_, pH 9.5, followed by development of the color reaction using nitroblue tetrazolium chloride/5-bromo-4-chloro-3-indolyl phosphate (1∶50; Roche Applied Science) in the previous solution. Sections were rinsed extensively in PBS and mounted with Fluoromount-G. Images were captured using a Retiga EXi Camera mounted on a Zeiss Axio Imager.M1 microscope and Northern Eclipse software.

## Supporting Information

Figure S1 β-gal activity and Nissl staining in the dorsolateral rostral hindbrain of adult *Runx1* ^lacZ/+^ mice. Coronal sections subjected to staining with X-gal (A) or Nissl substance (B) show the approximate region of β-gal activity in a triangular group of fairly dense cells. Scale bar  = 100 µm.
(1.55 MB TIF)

Figure S2 Expression of β-gal and TIP39 in the rostral hindbrain of E18.5 *Runx1* ^lacZ/+^ mouse embryos. In the sagittal plane, the TIP39^+^ cells of the MPL are located rostroventral to the group of β-gal+ cells. Abbreviation: MPL, medial paralemniscal nucleus. Scale bars  = 50 µm.
(2.26 MB TIF)

Figure S3 Expression of β-gal, VGLUT2 and MAP2 in the dorsolateral rostral hindbrain of E18.5 *Runx1* ^lacZ/+^ mouse embryos. Triple-label immunofluorescence staining of coronal sections for VGLUT2 (A), β-gal (B) and MAP2 (C) is shown merged in (D). β-gal^+^ neurons are located within a region of VGLUT2 immunoreactivity. Scale bar  = 10 µm.
(1.31 MB TIF)
