# Supplementary figures and images for: Molecular Characterization of the Mouse Superior Lateral Parabrachial Nucleus through Expression of the Transcription Factor Runx1
Source: PLoS One. 2010 Nov 11;5(11):e13944. doi: 10.1371/journal.pone.0013944 (PMC2978708; doi:10.1371/journal.pone.0013944)

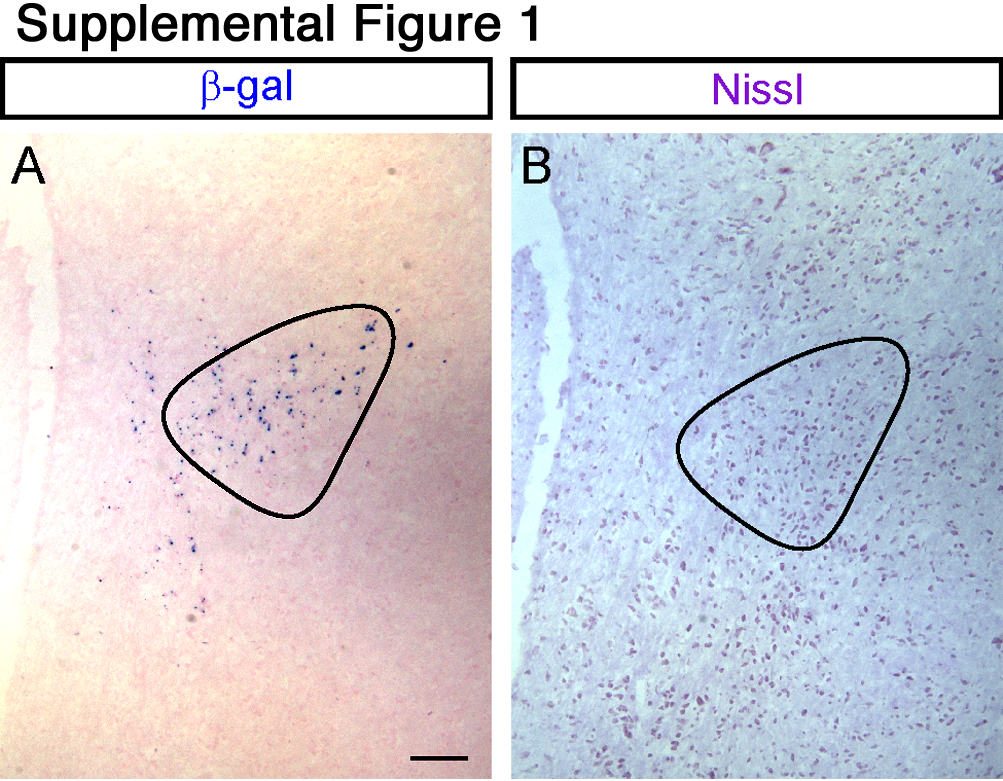

Supplement: Figure S1 — β-gal activity and Nissl staining in the dorsolateral rostral hindbrain of adult Runx1 lacZ/+ mice. Coronal sections subjected to staining with X-gal (A) or Nissl substance (B) show the approximate region of β-gal activity in a triangular group of fairly dense cells. Scale bar = 100 µm. (1.55 MB TIF) [file pone.0013944.s001.tif]

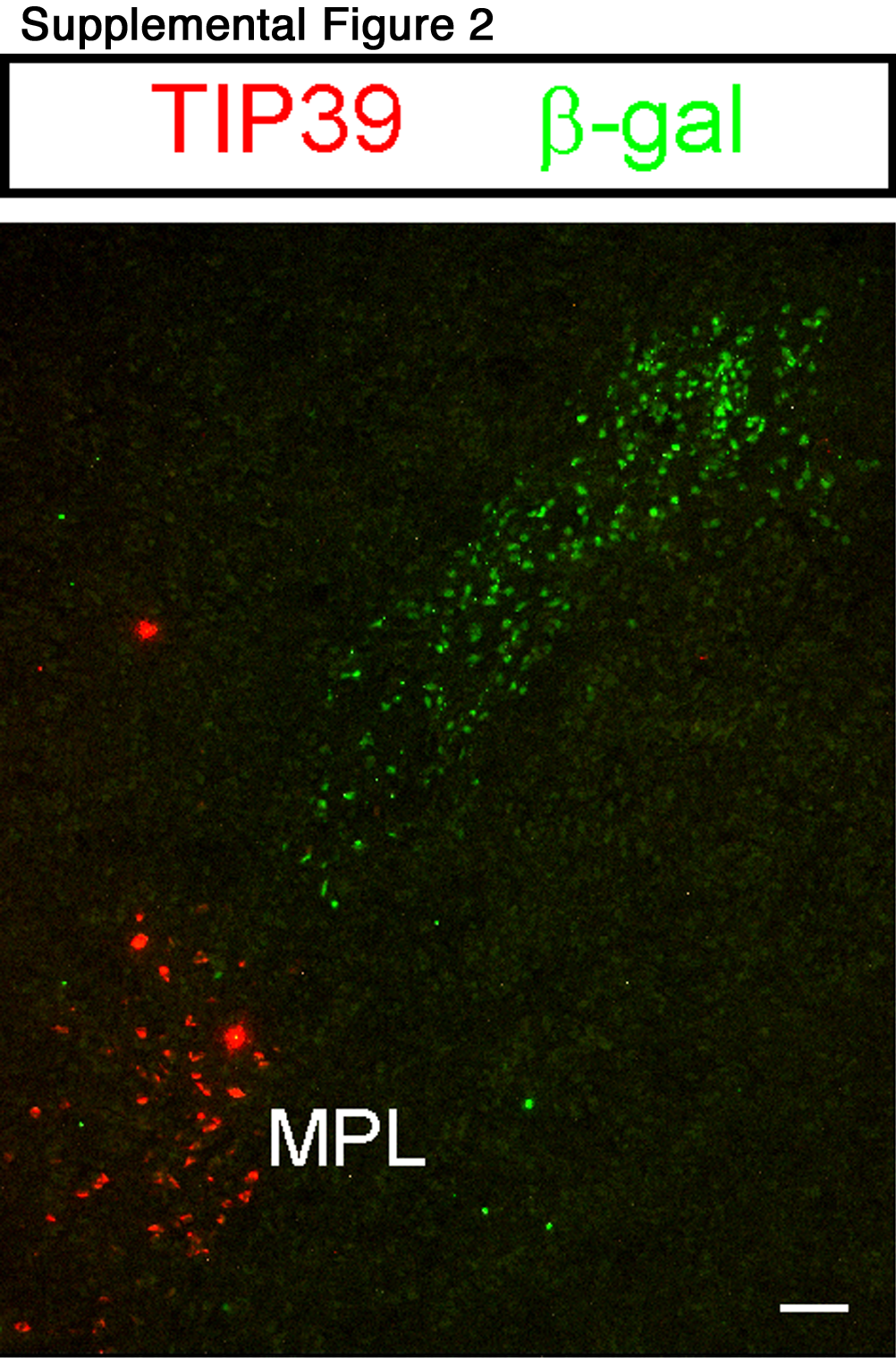

Supplement: Figure S2 — Expression of β-gal and TIP39 in the rostral hindbrain of E18.5 Runx1 lacZ/+ mouse embryos. In the sagittal plane, the TIP39+ cells of the MPL are located rostroventral to the group of β-gal+ cells. Abbreviation: MPL, medial paralemniscal nucleus. Scale bars = 50 µm. (2.26 MB TIF) [file pone.0013944.s002.tif]

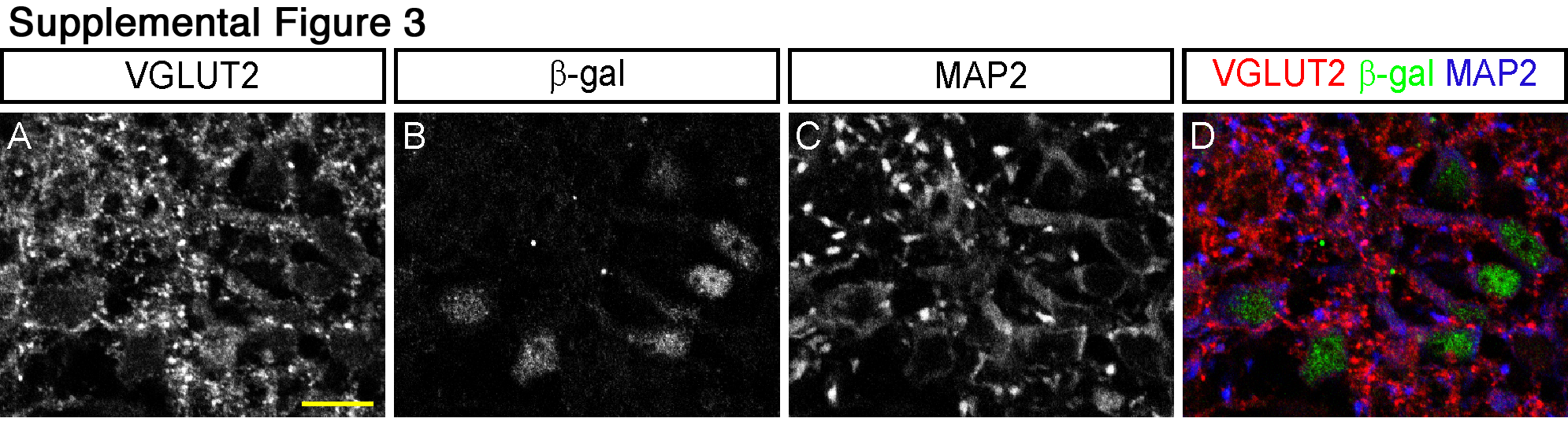

Supplement: Figure S3 — Expression of β-gal, VGLUT2 and MAP2 in the dorsolateral rostral hindbrain of E18.5 Runx1 lacZ/+ mouse embryos. Triple-label immunofluorescence staining of coronal sections for VGLUT2 (A), β-gal (B) and MAP2 (C) is shown merged in (D). β-gal+ neurons are located within a region of VGLUT2 immunoreactivity. Scale bar = 10 µm. (1.31 MB TIF) [file pone.0013944.s003.tif]
